# (*E*)-2-[4-(Diethyl­amino)­styr­yl]-1-methyl­pyridinium 4-chloro­benzene­sulfonate monohydrate

**DOI:** 10.1107/S1600536811034258

**Published:** 2011-08-27

**Authors:** Hoong-Kun Fun, Narissara Kaewmanee, Kullapa Chanawanno, Chatchanok Karalai, Suchada Chantrapromma

**Affiliations:** a X-ray Crystallography Unit, School of Physics, Universiti Sains Malaysia, 11800 USM, Penang, Malaysia; b Crystal Materials Research Unit, Department of Chemistry, Faculty of Science, Prince of Songkla University, Hat-Yai, Songkhla 90112, Thailand

## Abstract

In the title hydrated mol­ecular salt, C_18_H_23_N_2_^+^·C_6_H_4_ClO_3_S^−^·H_2_O, which shows moderate biological activity against methicillin-resistant *Staphylococcus aureus* (MRSA), one ethyl group of the 2-[4-(diethyl­amino)­styr­yl]-1-methyl­pyridinium cation is disordered over two orientations in a 0.604 (13):0.396 (13) ratio. The main part of the cation is nearly planar with a dihedral angle of 4.50 (10)° between the pyridinium and benzene rings. In the crystal, the components are linked by O—H⋯O hydrogen bonds and C—H⋯O weak inter­actions. Aromatic π–π stacking inter­actions with centroid–centroid separations of 3.7363 (12) and 3.7490 (13) Å also occur.

## Related literature

For background to and the application of quarternary ammonium compounds as disinfecta­nts, see: Brown & Skurray (2001 ▶); Chanawanno, Chantrapromma, Anantapong, Kanjana-Opas & Fun (2010 ▶); Domagk (1935 ▶); Endo *et al.* (1987 ▶); Fun *et al.* (2011 ▶); Wainwright & Kristiansen (2003 ▶). For a related structure, see: Fun *et al.* (2011 ▶); Kaewmanee *et al.* (2010 ▶). For the synthesis, see: Chanawanno, Chantrapromma, Anantapong & Kanjana-Opas (2010 ▶). For reference bond lengths, see: Allen *et al.* (1987 ▶).
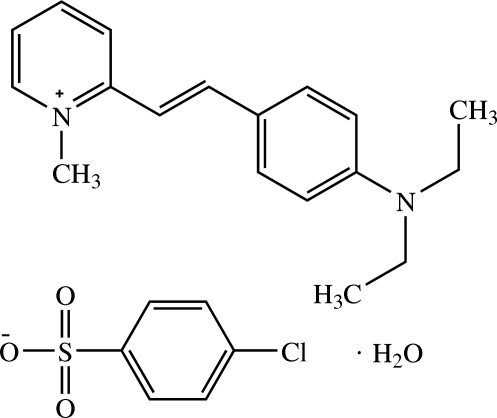


## Experimental

### 

#### Crystal data

C_18_H_23_N_2_^+^·C_6_H_4_ClO_3_S^−^·H_2_O
*M* *_r_* = 477.00
Triclinic, 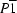

*a* = 7.2511 (3) Å
*b* = 10.2272 (4) Å
*c* = 16.7169 (7) Å
α = 88.441 (3)°
β = 80.057 (2)°
γ = 77.062 (2)°
*V* = 1190.00 (8) Å^3^
*Z* = 2
Mo *K*α radiation
μ = 0.28 mm^−1^
*T* = 100 K
0.53 × 0.25 × 0.04 mm

#### Data collection

Bruker APEX Duo CCD diffractometer
Absorption correction: multi-scan (*SADABS*; Bruker, 2009 ▶) *T*_min_ = 0.866, *T*_max_ = 0.990
15554 measured reflections
4617 independent reflections
3369 reflections with *I* > 2σ(*I*)
*R*_int_ = 0.031

#### Refinement

*R*[*F* ^2^ > 2σ(*F* ^2^)] = 0.044
*wR*(*F* ^2^) = 0.118
*S* = 1.04
4617 reflections
320 parameters
H atoms treated by a mixture of independent and constrained refinement
Δρ_max_ = 0.23 e Å^−3^
Δρ_min_ = −0.32 e Å^−3^

### 

Data collection: *APEX2* (Bruker, 2009 ▶); cell refinement: *SAINT* (Bruker, 2009 ▶); data reduction: *SAINT*; program(s) used to solve structure: *SHELXTL* (Sheldrick, 2008 ▶); program(s) used to refine structure: *SHELXTL*; molecular graphics: *SHELXTL*; software used to prepare material for publication: *SHELXTL* and *PLATON* (Spek, 2009 ▶).

## Supplementary Material

Crystal structure: contains datablock(s) global, I. DOI: 10.1107/S1600536811034258/hb6382sup1.cif

Structure factors: contains datablock(s) I. DOI: 10.1107/S1600536811034258/hb6382Isup2.hkl

Supplementary material file. DOI: 10.1107/S1600536811034258/hb6382Isup3.cml

Additional supplementary materials:  crystallographic information; 3D view; checkCIF report

## Figures and Tables

**Table 1:** Hydrogen-bond geometry (Å, °)

*D*—H⋯*A*	*D*—H	H⋯*A*	*D*⋯*A*	*D*—H⋯*A*
O1*W*—H2*W*1⋯O1	0.81 (3)	1.98 (3)	2.783 (3)	174 (3)
O1*W*—H1*W*1⋯O2^i^	0.87 (3)	2.13 (4)	2.977 (3)	166 (3)
C2—H2*A*⋯O2^ii^	0.93	2.52	3.374 (3)	153
C4—H4*A*⋯O1*W*^iii^	0.93	2.43	3.316 (3)	158
C13—H13*A*⋯O3	0.93	2.59	3.495 (3)	164
C18—H18*A*⋯O2^iv^	0.96	2.49	3.426 (3)	166
C18—H18*C*⋯O3	0.96	2.57	3.202 (3)	123

Symmetry codes: (i) 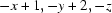; (ii) 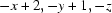; (iii) 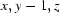; (iv) 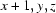.

**Fig. 1.**
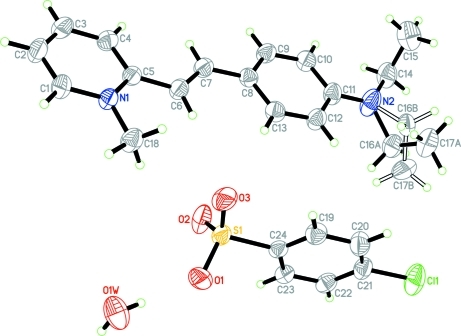
The asymmetric unit of (I) showing 40% probability displacement ellipsoids. Open bonds show the minor component.

**Fig. 2.**
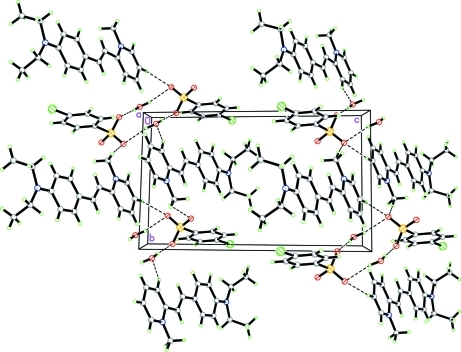
The crystal packing of the major component viewed along the b axis. The O—H···O hydrogen bonds and weak C—H···O interactions are drawn as dashed lines.

## supplementary crystallographic information

### Comment

As disinfectants, quaternary ammonium compounds (QACs) have been used for hygienic care in both medical and domestic purposes due to their low toxicity and wide-ranging antimicrobial properties for a long time (Domagk, 1935). However, the long-term use of any disinfectants will lead to the resistance phenomena of some bacterial strains that makes these disinfectants to become unpractical for real life usage. The appearance of resistant microganisms against QACs, especially Methicillin-resistant *Staphylococcus aureus* (MRSA), made the common QACs such as benzalkonium chloride and cetylpridinium chloride to be inadequate for MRSA treatment (Wainwright & Kristiansen, 2003; Brown & Skurray, 2001). Therefore, we decided to develop the novel pyridinium QACs which were expected to overcome this Staphylococcus-resistant phenomenon by modifying the QACs structures and to study their anti-MRSA activity. Among various chromophores employed in the research for chemotherapeutic drug design, tertiary amine seems to be an interesting group to be introduced into the structure (Endo *et al.*, 1987). The title compound (I) was one among many pyridinium QACs synthesized in our laboratory (Chanawanno, Chantrapromma, Anantapong, Kanjana-Opas & Fun, 2010) hoping for a new antibacterial drug candidate and this compound showed moderate activity against MRSA with the MIC value of 150 mg/ml. Herein its crystal structure is reported.

The asymmetric unit of the title compound (I) (Fig. 1) consists of the C_18_H_23_N_2_^+^ cation, C_6_H_4_ClO_3_S^-^ anion and one H_2_O molecule. The cation exists in the *E* configuration with respect to the C6═C7 double bond [1.337 (3) Å]. The cation is nearly planar with the the dihedral angle between the C1–C5/N1 pyridinium and the C8–C13 benzene rings being 4.50 (10)° and the torsion angle C5–C6–C7–C8 = 177.3 (2)°. One ethyl unit of the diethylamino moiety is disordered over two orientations; the major component *A* and the minor component *B* (Fig. 1), with the refined site-occupancy ratio of 0.604 (13)/0.396 (13). The diethylamino moiety is deviated from the attached benzene ring. Its conformation can be indicated by the torsion angles C11–N2–C14–C15 = 78.6 (3)°, C11–N2–C16–C17 = -95.0 (4)° for the major component *A* and 107.1 (5)° for the minor component *B*. The cation and anion are inclined to each other as indicated by the dihedral angles between the pyridinium and benzene rings of cation, and the sulfonate substituted benzene ring being 83.96 (10) and 86.97 (11)°, respectively. The bond lengths are in normal ranges (Allen *et al.*, 1987) and comparable with a related structures (Fun *et al.*, 2011; Kaewmanee *et al.*, 2010).

In the crystal packing, the cations, anions and water molecules are linked into a network by O—H..O hydrogen bonds and C—H···O weak interactions (Fig. 2 and Table 1). π···π interactions with the centroid distances of Cg_1_···Cg_1_^ii^ = 3.7363 (12) Å and Cg_1_···Cg_2_^iv^ = 3.7490 (13) Å were observed; Cg_1_ and Cg_2_ are the centroids of N1/C1–C5 and C8–C13 rings, respectively.

### Experimental

(*E*)-2-(4-(diethylamino)styryl)-1-methylpyridinium iodide (compound A, 0.13 g, 0.33 mmol) was prepared by the previous method (Kaewmanee *et al.*, 2010) and then was mixed with silver (I) 4-chlorobenzenesulfonate (Chanawanno, Chantrapromma, Anantapong & Kanjana-Opas, 2010) (0.10 g, 0.33 mmol) in methanol (100 ml). The mixture immediately yielded a grey precipitate of silver iodide. After stirring the mixture for 30 min, the precipitate of silver iodide was removed and the resulting solution was evaporated yielding an orange solid of the title compound. Orange plates of (I) were recrystallized from methanol by slow evaporation of the solvent at room temperature after a few weeks, Mp. 446-448 K.

### Refinement

Water H atoms were located in difference maps and refined isotropically. The remaining H atoms were placed in calculated positions with d(C—H) = 0.93 Å, *U*_iso_=1.2*U*_eq_(C) for aromatic and CH and 0.96 Å, *U*_iso_ = 1.5*U*_eq_(C) for CH_3_ atoms. A rotating group model was used for the methyl groups. The highest residual electron density peak is located at 0.91 Å from O1 and the deepest hole is located at 0.71 Å from S1.

### Crystal data

**Table:**

C_18_H_23_N_2_^+^·C_6_H_4_ClO_3_S^−^·H_2_O	*Z* = 2
*M**_r_* = 477.00	*F*(000) = 504
Triclinic, *P*1	*D*_x_ = 1.331 Mg m^−^^3^
Hall symbol: -P 1	Melting point = 446–448 K
*a* = 7.2511 (3) Å	Mo *K*α radiation, λ = 0.71073 Å
*b* = 10.2272 (4) Å	Cell parameters from 4617 reflections
*c* = 16.7169 (7) Å	θ = 2.0–26.0°
α = 88.441 (3)°	µ = 0.28 mm^−^^1^
β = 80.057 (2)°	*T* = 296 K
γ = 77.062 (2)°	Plate, orange
*V* = 1190.00 (8) Å^3^	0.53 × 0.25 × 0.04 mm

### Data collection

**Table:**

Bruker APEX Duo CCD diffractometer	4617 independent reflections
Radiation source: sealed tube	3369 reflections with *I* > 2σ(*I*)
graphite	*R*_int_ = 0.031
φ and ω scans	θ_max_ = 26.0°, θ_min_ = 2.0°
Absorption correction: multi-scan (*SADABS*; Bruker, 2009)	*h* = −8→8
*T*_min_ = 0.866, *T*_max_ = 0.990	*k* = −12→12
15554 measured reflections	*l* = −20→20

### Refinement

**Table:**

Refinement on *F*^2^	Primary atom site location: structure-invariant direct methods
Least-squares matrix: full	Secondary atom site location: difference Fourier map
*R*[*F*^2^ > 2σ(*F*^2^)] = 0.044	Hydrogen site location: inferred from neighbouring sites
*wR*(*F*^2^) = 0.118	H atoms treated by a mixture of independent and constrained refinement
*S* = 1.04	*w* = 1/[σ^2^(*F*_o_^2^) + (0.0473*P*)^2^ + 0.3949*P*] where *P* = (*F*_o_^2^ + 2*F*_c_^2^)/3
4617 reflections	(Δ/σ)_max_ = 0.001
320 parameters	Δρ_max_ = 0.23 e Å^−^^3^
0 restraints	Δρ_min_ = −0.32 e Å^−^^3^

### Special details

**Table:**

Geometry. All esds (except the esd in the dihedral angle between two l.s. planes) are estimated using the full covariance matrix. The cell esds are taken into account individually in the estimation of esds in distances, angles and torsion angles; correlations between esds in cell parameters are only used when they are defined by crystal symmetry. An approximate (isotropic) treatment of cell esds is used for estimating esds involving l.s. planes.
Refinement. Refinement of F^2^ against ALL reflections. The weighted R-factor wR and goodness of fit S are based on F^2^, conventional R-factors R are based on F, with F set to zero for negative F^2^. The threshold expression of F^2^ > 2sigma(F^2^) is used only for calculating R-factors(gt) etc. and is not relevant to the choice of reflections for refinement. R-factors based on F^2^ are statistically about twice as large as those based on F, and R- factors based on ALL data will be even larger.

### Fractional atomic coordinates and isotropic or equivalent isotropic displacement parameters (Å^2^)

**Table:**

	*x*	*y*	*z*	*U*_iso_*/*U*_eq_	Occ. (<1)
Cl1	−0.43901 (10)	1.01358 (9)	0.38727 (5)	0.0885 (3)	
S1	0.36666 (7)	0.85852 (5)	0.16430 (4)	0.04745 (17)	
O1	0.4113 (2)	0.98202 (16)	0.13126 (11)	0.0731 (5)	
O2	0.3361 (2)	0.77323 (18)	0.10248 (11)	0.0719 (5)	
O3	0.4986 (2)	0.79002 (17)	0.21510 (11)	0.0669 (5)	
N1	1.0111 (2)	0.50652 (16)	0.11414 (10)	0.0417 (4)	
N2	−0.1490 (3)	0.4451 (2)	0.37633 (15)	0.0787 (7)	
C1	1.1941 (3)	0.4789 (2)	0.07236 (13)	0.0502 (5)	
H1A	1.2646	0.5451	0.0676	0.060*	
C2	1.2759 (3)	0.3571 (2)	0.03751 (14)	0.0570 (6)	
H2A	1.4011	0.3395	0.0091	0.068*	
C3	1.1701 (4)	0.2594 (2)	0.04490 (14)	0.0587 (6)	
H3A	1.2239	0.1750	0.0215	0.070*	
C4	0.9856 (3)	0.2870 (2)	0.08674 (14)	0.0521 (6)	
H4A	0.9146	0.2210	0.0908	0.063*	
C5	0.9018 (3)	0.4124 (2)	0.12341 (12)	0.0413 (5)	
C6	0.7102 (3)	0.4465 (2)	0.17040 (13)	0.0471 (5)	
H6A	0.6627	0.5346	0.1889	0.056*	
C7	0.5968 (3)	0.3595 (2)	0.18895 (13)	0.0478 (5)	
H7A	0.6453	0.2731	0.1678	0.057*	
C8	0.4073 (3)	0.3844 (2)	0.23826 (12)	0.0430 (5)	
C9	0.3068 (3)	0.2820 (2)	0.25040 (14)	0.0506 (5)	
H9A	0.3640	0.1985	0.2263	0.061*	
C10	0.1273 (3)	0.2995 (2)	0.29644 (14)	0.0518 (5)	
H10A	0.0667	0.2279	0.3035	0.062*	
C11	0.0335 (3)	0.4238 (2)	0.33314 (14)	0.0524 (6)	
C12	0.1351 (3)	0.5271 (2)	0.32114 (14)	0.0530 (6)	
H12A	0.0785	0.6107	0.3451	0.064*	
C13	0.3153 (3)	0.5076 (2)	0.27510 (13)	0.0479 (5)	
H13A	0.3774	0.5785	0.2684	0.057*	
C14	−0.2465 (4)	0.3354 (3)	0.39602 (17)	0.0684 (7)	
H14A	−0.2227	0.2776	0.3486	0.082*	
H14B	−0.3838	0.3722	0.4087	0.082*	
C15	−0.1841 (5)	0.2521 (3)	0.46642 (18)	0.0881 (9)	
H15A	−0.2525	0.1814	0.4758	0.132*	
H15B	−0.2114	0.3078	0.5142	0.132*	
H15C	−0.0487	0.2141	0.4541	0.132*	
C18	0.9351 (3)	0.6428 (2)	0.14925 (15)	0.0536 (6)	
H18A	1.0313	0.6947	0.1363	0.080*	
H18B	0.9016	0.6372	0.2072	0.080*	
H18C	0.8231	0.6852	0.1271	0.080*	
C19	0.1250 (3)	0.8958 (2)	0.31157 (14)	0.0518 (6)	
H19A	0.2346	0.8653	0.3346	0.062*	
C20	−0.0530 (4)	0.9297 (3)	0.36085 (14)	0.0598 (6)	
H20A	−0.0638	0.9228	0.4170	0.072*	
C21	−0.2136 (3)	0.9736 (2)	0.32577 (14)	0.0526 (6)	
C22	−0.2011 (3)	0.9859 (2)	0.24371 (14)	0.0505 (5)	
H22A	−0.3114	1.0157	0.2210	0.061*	
C23	−0.0230 (3)	0.9535 (2)	0.19461 (13)	0.0446 (5)	
H23A	−0.0127	0.9629	0.1386	0.053*	
C24	0.1407 (3)	0.90710 (19)	0.22881 (12)	0.0400 (5)	
O1W	0.7231 (3)	1.0760 (3)	0.04924 (15)	0.0793 (6)	
C16A	−0.2701 (8)	0.5883 (8)	0.3938 (4)	0.0633 (19)	0.604 (13)
H16A	−0.2270	0.6490	0.3529	0.076*	0.604 (13)
H16B	−0.4041	0.5906	0.3928	0.076*	0.604 (13)
C17A	−0.2473 (8)	0.6301 (8)	0.4756 (4)	0.084 (2)	0.604 (13)
H17A	−0.3215	0.7197	0.4877	0.125*	0.604 (13)
H17B	−0.1143	0.6275	0.4761	0.125*	0.604 (13)
H17C	−0.2914	0.5700	0.5157	0.125*	0.604 (13)
C16B	−0.2117 (11)	0.5539 (10)	0.4408 (6)	0.054 (3)	0.396 (13)
H16C	−0.1036	0.5870	0.4524	0.065*	0.396 (13)
H16D	−0.2780	0.5229	0.4906	0.065*	0.396 (13)
C17B	−0.3475 (16)	0.6607 (11)	0.3998 (6)	0.084 (3)	0.396 (13)
H17D	−0.4024	0.7355	0.4363	0.126*	0.396 (13)
H17E	−0.4482	0.6232	0.3860	0.126*	0.396 (13)
H17F	−0.2772	0.6904	0.3513	0.126*	0.396 (13)
H2W1	0.630 (4)	1.048 (3)	0.0698 (17)	0.073 (10)*	
H1W1	0.697 (5)	1.109 (4)	0.003 (2)	0.111 (14)*	

### Atomic displacement parameters (Å^2^)

**Table:**

	*U*^11^	*U*^22^	*U*^33^	*U*^12^	*U*^13^	*U*^23^
Cl1	0.0520 (4)	0.1114 (6)	0.0905 (5)	−0.0164 (4)	0.0210 (4)	−0.0234 (4)
S1	0.0366 (3)	0.0405 (3)	0.0620 (4)	−0.0105 (2)	0.0036 (2)	−0.0030 (2)
O1	0.0612 (11)	0.0534 (10)	0.0986 (14)	−0.0201 (8)	0.0104 (10)	0.0177 (9)
O2	0.0571 (10)	0.0803 (12)	0.0747 (11)	−0.0242 (9)	0.0155 (9)	−0.0323 (10)
O3	0.0387 (9)	0.0646 (11)	0.0905 (12)	0.0004 (8)	−0.0082 (8)	0.0063 (9)
N1	0.0351 (9)	0.0417 (9)	0.0464 (9)	−0.0070 (7)	−0.0027 (7)	−0.0046 (8)
N2	0.0593 (13)	0.0663 (14)	0.1036 (18)	−0.0285 (11)	0.0279 (12)	−0.0248 (13)
C1	0.0364 (11)	0.0580 (14)	0.0547 (13)	−0.0115 (10)	−0.0021 (10)	−0.0019 (11)
C2	0.0392 (12)	0.0628 (15)	0.0600 (14)	−0.0004 (11)	0.0026 (10)	−0.0057 (12)
C3	0.0592 (15)	0.0479 (13)	0.0583 (14)	0.0043 (12)	−0.0002 (12)	−0.0084 (11)
C4	0.0539 (14)	0.0397 (12)	0.0587 (13)	−0.0075 (10)	−0.0017 (11)	−0.0023 (10)
C5	0.0395 (11)	0.0400 (11)	0.0436 (11)	−0.0082 (9)	−0.0054 (9)	0.0000 (9)
C6	0.0431 (12)	0.0415 (12)	0.0536 (12)	−0.0091 (10)	0.0005 (10)	−0.0050 (10)
C7	0.0452 (12)	0.0420 (12)	0.0539 (13)	−0.0082 (10)	−0.0037 (10)	−0.0012 (10)
C8	0.0431 (12)	0.0425 (12)	0.0450 (11)	−0.0137 (9)	−0.0063 (9)	0.0016 (9)
C9	0.0491 (13)	0.0403 (12)	0.0606 (14)	−0.0105 (10)	−0.0028 (11)	−0.0044 (10)
C10	0.0504 (13)	0.0477 (13)	0.0600 (14)	−0.0215 (10)	−0.0023 (11)	−0.0025 (11)
C11	0.0462 (13)	0.0554 (14)	0.0549 (13)	−0.0182 (11)	0.0036 (10)	−0.0045 (11)
C12	0.0531 (13)	0.0450 (13)	0.0584 (13)	−0.0135 (11)	0.0026 (11)	−0.0099 (10)
C13	0.0497 (13)	0.0445 (12)	0.0521 (12)	−0.0193 (10)	−0.0041 (10)	−0.0009 (10)
C14	0.0514 (14)	0.0794 (18)	0.0754 (17)	−0.0292 (13)	0.0069 (13)	−0.0073 (14)
C15	0.094 (2)	0.103 (2)	0.0773 (19)	−0.0458 (19)	−0.0084 (17)	−0.0045 (18)
C18	0.0492 (13)	0.0458 (12)	0.0646 (14)	−0.0124 (10)	−0.0019 (11)	−0.0135 (11)
C19	0.0433 (12)	0.0552 (14)	0.0551 (13)	−0.0051 (10)	−0.0108 (10)	0.0004 (11)
C20	0.0592 (15)	0.0694 (16)	0.0466 (13)	−0.0098 (12)	−0.0026 (11)	−0.0033 (11)
C21	0.0419 (12)	0.0516 (13)	0.0603 (14)	−0.0114 (10)	0.0056 (11)	−0.0100 (11)
C22	0.0389 (12)	0.0472 (13)	0.0642 (15)	−0.0049 (10)	−0.0103 (11)	−0.0056 (11)
C23	0.0425 (12)	0.0415 (11)	0.0488 (12)	−0.0068 (9)	−0.0085 (10)	−0.0013 (9)
C24	0.0375 (11)	0.0299 (10)	0.0519 (12)	−0.0098 (8)	−0.0026 (9)	−0.0027 (9)
O1W	0.0702 (14)	0.1094 (18)	0.0700 (14)	−0.0476 (13)	−0.0091 (11)	0.0081 (12)
C16A	0.042 (3)	0.073 (5)	0.070 (4)	−0.010 (3)	0.000 (3)	0.000 (3)
C17A	0.077 (4)	0.090 (5)	0.076 (4)	−0.013 (3)	0.004 (3)	−0.024 (4)
C16B	0.049 (4)	0.064 (6)	0.048 (5)	−0.015 (4)	0.005 (3)	0.000 (4)
C17B	0.072 (6)	0.061 (6)	0.102 (7)	0.012 (5)	0.001 (5)	0.003 (5)

### Geometric parameters (Å, °)

**Table:**

Cl1—C21	1.743 (2)	C13—H13A	0.9300
S1—O3	1.4406 (17)	C14—C15	1.507 (4)
S1—O1	1.4453 (16)	C14—H14A	0.9700
S1—O2	1.4466 (17)	C14—H14B	0.9700
S1—C24	1.775 (2)	C15—H15A	0.9600
N1—C1	1.361 (3)	C15—H15B	0.9600
N1—C5	1.367 (3)	C15—H15C	0.9600
N1—C18	1.477 (3)	C18—H18A	0.9600
N2—C11	1.367 (3)	C18—H18B	0.9600
N2—C14	1.456 (3)	C18—H18C	0.9600
N2—C16B	1.508 (11)	C19—C24	1.372 (3)
N2—C16A	1.537 (8)	C19—C20	1.383 (3)
C1—C2	1.354 (3)	C19—H19A	0.9300
C1—H1A	0.9300	C20—C21	1.375 (3)
C2—C3	1.381 (3)	C20—H20A	0.9300
C2—H2A	0.9300	C21—C22	1.363 (3)
C3—C4	1.370 (3)	C22—C23	1.382 (3)
C3—H3A	0.9300	C22—H22A	0.9300
C4—C5	1.398 (3)	C23—C24	1.389 (3)
C4—H4A	0.9300	C23—H23A	0.9300
C5—C6	1.446 (3)	O1W—H2W1	0.81 (3)
C6—C7	1.337 (3)	O1W—H1W1	0.86 (4)
C6—H6A	0.9300	C16A—C17A	1.490 (11)
C7—C8	1.448 (3)	C16A—H16A	0.9700
C7—H7A	0.9300	C16A—H16B	0.9700
C8—C13	1.391 (3)	C17A—H17A	0.9600
C8—C9	1.397 (3)	C17A—H17B	0.9600
C9—C10	1.370 (3)	C17A—H17C	0.9600
C9—H9A	0.9300	C16B—C17B	1.531 (15)
C10—C11	1.402 (3)	C16B—H16C	0.9700
C10—H10A	0.9300	C16B—H16D	0.9700
C11—C12	1.408 (3)	C17B—H17D	0.9600
C12—C13	1.372 (3)	C17B—H17E	0.9600
C12—H12A	0.9300	C17B—H17F	0.9600
			
O3—S1—O1	113.45 (11)	N2—C14—H14A	108.8
O3—S1—O2	113.60 (11)	C15—C14—H14A	108.8
O1—S1—O2	112.13 (12)	N2—C14—H14B	108.8
O3—S1—C24	106.07 (10)	C15—C14—H14B	108.8
O1—S1—C24	105.37 (10)	H14A—C14—H14B	107.7
O2—S1—C24	105.31 (9)	C14—C15—H15A	109.5
C1—N1—C5	121.61 (18)	C14—C15—H15B	109.5
C1—N1—C18	117.11 (18)	H15A—C15—H15B	109.5
C5—N1—C18	121.27 (17)	C14—C15—H15C	109.5
C11—N2—C14	121.6 (2)	H15A—C15—H15C	109.5
C11—N2—C16B	118.8 (3)	H15B—C15—H15C	109.5
C14—N2—C16B	111.8 (3)	N1—C18—H18A	109.5
C11—N2—C16A	120.6 (3)	N1—C18—H18B	109.5
C14—N2—C16A	117.1 (3)	H18A—C18—H18B	109.5
C2—C1—N1	121.5 (2)	N1—C18—H18C	109.5
C2—C1—H1A	119.3	H18A—C18—H18C	109.5
N1—C1—H1A	119.3	H18B—C18—H18C	109.5
C1—C2—C3	118.8 (2)	C24—C19—C20	120.3 (2)
C1—C2—H2A	120.6	C24—C19—H19A	119.9
C3—C2—H2A	120.6	C20—C19—H19A	119.9
C4—C3—C2	119.9 (2)	C21—C20—C19	119.1 (2)
C4—C3—H3A	120.1	C21—C20—H20A	120.4
C2—C3—H3A	120.1	C19—C20—H20A	120.4
C3—C4—C5	121.3 (2)	C22—C21—C20	121.5 (2)
C3—C4—H4A	119.3	C22—C21—Cl1	119.02 (18)
C5—C4—H4A	119.3	C20—C21—Cl1	119.46 (19)
N1—C5—C4	116.93 (19)	C21—C22—C23	119.3 (2)
N1—C5—C6	119.05 (18)	C21—C22—H22A	120.4
C4—C5—C6	124.0 (2)	C23—C22—H22A	120.4
C7—C6—C5	124.14 (19)	C22—C23—C24	120.0 (2)
C7—C6—H6A	117.9	C22—C23—H23A	120.0
C5—C6—H6A	117.9	C24—C23—H23A	120.0
C6—C7—C8	127.4 (2)	C19—C24—C23	119.73 (19)
C6—C7—H7A	116.3	C19—C24—S1	120.97 (16)
C8—C7—H7A	116.3	C23—C24—S1	119.28 (16)
C13—C8—C9	116.57 (19)	H2W1—O1W—H1W1	105 (3)
C13—C8—C7	123.52 (19)	C17A—C16A—N2	108.1 (7)
C9—C8—C7	119.92 (19)	C17A—C16A—H16A	110.1
C10—C9—C8	122.6 (2)	N2—C16A—H16A	110.1
C10—C9—H9A	118.7	C17A—C16A—H16B	110.1
C8—C9—H9A	118.7	N2—C16A—H16B	110.1
C9—C10—C11	121.0 (2)	H16A—C16A—H16B	108.4
C9—C10—H10A	119.5	N2—C16B—C17B	101.3 (8)
C11—C10—H10A	119.5	N2—C16B—H16C	111.5
N2—C11—C10	121.9 (2)	C17B—C16B—H16C	111.5
N2—C11—C12	121.6 (2)	N2—C16B—H16D	111.5
C10—C11—C12	116.4 (2)	C17B—C16B—H16D	111.5
C13—C12—C11	121.8 (2)	H16C—C16B—H16D	109.3
C13—C12—H12A	119.1	C16B—C17B—H17D	109.5
C11—C12—H12A	119.1	C16B—C17B—H17E	109.5
C12—C13—C8	121.6 (2)	H17D—C17B—H17E	109.5
C12—C13—H13A	119.2	C16B—C17B—H17F	109.5
C8—C13—H13A	119.2	H17D—C17B—H17F	109.5
N2—C14—C15	113.7 (2)	H17E—C17B—H17F	109.5
			
C5—N1—C1—C2	0.5 (3)	C11—C12—C13—C8	−0.4 (3)
C18—N1—C1—C2	−179.5 (2)	C9—C8—C13—C12	0.1 (3)
N1—C1—C2—C3	0.0 (3)	C7—C8—C13—C12	179.7 (2)
C1—C2—C3—C4	0.2 (4)	C11—N2—C14—C15	78.6 (3)
C2—C3—C4—C5	−0.9 (3)	C16B—N2—C14—C15	−70.2 (5)
C1—N1—C5—C4	−1.1 (3)	C16A—N2—C14—C15	−111.0 (4)
C18—N1—C5—C4	178.96 (19)	C24—C19—C20—C21	0.5 (4)
C1—N1—C5—C6	178.28 (18)	C19—C20—C21—C22	−0.7 (4)
C18—N1—C5—C6	−1.7 (3)	C19—C20—C21—Cl1	178.81 (18)
C3—C4—C5—N1	1.3 (3)	C20—C21—C22—C23	−0.1 (3)
C3—C4—C5—C6	−178.1 (2)	Cl1—C21—C22—C23	−179.58 (17)
N1—C5—C6—C7	−174.3 (2)	C21—C22—C23—C24	1.0 (3)
C4—C5—C6—C7	5.0 (3)	C20—C19—C24—C23	0.4 (3)
C5—C6—C7—C8	177.3 (2)	C20—C19—C24—S1	−177.73 (18)
C6—C7—C8—C13	−0.7 (4)	C22—C23—C24—C19	−1.2 (3)
C6—C7—C8—C9	178.9 (2)	C22—C23—C24—S1	177.00 (15)
C13—C8—C9—C10	−0.4 (3)	O3—S1—C24—C19	9.9 (2)
C7—C8—C9—C10	180.0 (2)	O1—S1—C24—C19	−110.69 (19)
C8—C9—C10—C11	0.9 (4)	O2—S1—C24—C19	130.60 (19)
C14—N2—C11—C10	8.5 (4)	O3—S1—C24—C23	−168.25 (16)
C16B—N2—C11—C10	155.2 (4)	O1—S1—C24—C23	71.18 (18)
C16A—N2—C11—C10	−161.5 (3)	O2—S1—C24—C23	−47.53 (19)
C14—N2—C11—C12	−173.5 (2)	C11—N2—C16A—C17A	−95.0 (4)
C16B—N2—C11—C12	−26.7 (5)	C14—N2—C16A—C17A	94.5 (4)
C16A—N2—C11—C12	16.5 (5)	C16B—N2—C16A—C17A	3.5 (5)
C9—C10—C11—N2	177.1 (2)	C11—N2—C16B—C17B	107.1 (5)
C9—C10—C11—C12	−1.1 (3)	C14—N2—C16B—C17B	−103.1 (5)
N2—C11—C12—C13	−177.3 (2)	C16A—N2—C16B—C17B	3.4 (5)
C10—C11—C12—C13	0.8 (3)		

### Hydrogen-bond geometry (Å, °)

**Table:**

*D*—H···*A*	*D*—H	H···*A*	*D*···*A*	*D*—H···*A*
O1W—H2W1···O1	0.81 (3)	1.98 (3)	2.783 (3)	174 (3)
O1W—H1W1···O2^i^	0.87 (3)	2.13 (4)	2.977 (3)	166 (3)
C2—H2A···O2^ii^	0.93	2.52	3.374 (3)	153
C4—H4A···O1W^iii^	0.93	2.43	3.316 (3)	158
C13—H13A···O3	0.93	2.59	3.495 (3)	164
C18—H18A···O2^iv^	0.96	2.49	3.426 (3)	166
C18—H18C···O3	0.96	2.57	3.202 (3)	123

Symmetry codes:  (i) −*x*+1, −*y*+2, −*z*; (ii) −*x*+2, −*y*+1, −*z*; (iii) *x*, *y*−1, *z*; (iv) *x*+1, *y*, *z*.

## References

[bb1] Allen, F. H., Kennard, O., Watson, D. G., Brammer, L., Orpen, A. G. & Taylor, R. (1987). *J. Chem. Soc. Perkin Trans. 2*, pp. S1–S19.

[bb2] Brown, M. H. & Skurray, R. A. (2001). *J. Mol. Microbiol. Biotechnol.* **3**, 163–170. 11321569

[bb3] Bruker (2009). *APEX2*, *SAINT* and *SADABS* Bruker AXS Inc., Madison, Wisconsin, USA.

[bb4] Chanawanno, K., Chantrapromma, S., Anantapong, T. & Kanjana-Opas, A. (2010). *Lat. Am. J. Pharm* **29**, 1166–1170.

[bb5] Chanawanno, K., Chantrapromma, S., Anantapong, T., Kanjana-Opas, A. & Fun, H.-K. (2010). *Eur. J. Med. Chem.* **45**, 4199-4208. 10.1016/j.ejmech.2010.06.014 20619939

[bb6] Domagk, G. (1935). *Dtsch Med. Wochenschr.* **24**, 829–832.

[bb7] Endo, Y., Tani, T. & Kodama, K. (1987). *Appl. Environ. Microbiol.* **53**, 2050–2055. 10.1128/aem.53.9.2050-2055.1987 PMC204056 3314703

[bb8] Fun, H.-K., Kaewmanee, N., Chanawanno, K. & Chantrapromma, S. (2011). *Acta Cryst.* E**67**, o593–o594. 10.1107/S1600536811004156 PMC3051968 21522353

[bb9] Kaewmanee, N., Chanawanno, K., Chantrapromma, S. & Fun, H.-K. (2010). *Acta Cryst.* E**66**, o2639–o2640. 10.1107/S1600536810037505 PMC2983428 21587611

[bb10] Sheldrick, G. M. (2008). *Acta Cryst.* A**64**, 112–122. 10.1107/S0108767307043930 18156677

[bb11] Spek, A. L. (2009). *Acta Cryst.* D**65**, 148–155. 10.1107/S090744490804362X PMC2631630 19171970

[bb12] Wainwright, M. & Kristiansen, J. E. (2003). *Int. J. Antimicrob. Agents*, **22**, 479–486. 10.1016/s0924-8579(03)00264-4 14602365

